# LIQ HD (Lick Instance Quantifier Home Cage Device): An Open-Source Tool for Recording Undisturbed Two-Bottle Drinking Behavior in a Home Cage Environment

**DOI:** 10.1523/ENEURO.0506-22.2023

**Published:** 2023-04-12

**Authors:** Nicholas Petersen, Danielle N. Adank, Ritika Raghavan, Danny G. Winder, Marie A. Doyle

**Affiliations:** 1 Vanderbilt Brain Institute, Vanderbilt University School of Medicine, Nashville, TN 37232; 2 Vanderbilt Center for Addiction Research, Vanderbilt University School of Medicine, Nashville, TN 37232; 3 Department of Molecular Physiology and Biophysics, Vanderbilt University School of Medicine, Nashville, TN 37232; 4 Department of Pharmacology, Vanderbilt University School of Medicine, Nashville, TN 37232

**Keywords:** bout microstructure, capacitive sensor, drinking behavior, lickometer, two-bottle choice

## Abstract

Investigation of rodent drinking behavior has provided insight into drivers of thirst, circadian rhythms, anhedonia, and drug and ethanol consumption. Traditional methods of recording fluid intake involve weighing bottles, which is cumbersome and lacks temporal resolution. Several open-source devices have been designed to improve drink monitoring, particularly for two-bottle choice tasks. However, beam-break sensors lack the ability to detect individual licks for bout microstructure analysis. Thus, we designed LIQ HD (Lick Instance Quantifier Home cage Device) with the goal of using capacitive sensors to increase accuracy and analyze lick microstructure, building a device compatible with ventilated home cages, increasing scale with prolonged undisturbed recordings, and creating a design that is easy to build and use with an intuitive touchscreen graphical user interface. The system tracks two-bottle choice licking behavior in up to 18 rodent cages, or 36 single bottles, on a minute-to-minute timescale controlled by a single Arduino microcontroller. The data are logged to a single SD card, allowing for efficient downstream analysis. LIQ HD accuracy was validated with sucrose, quinine, and ethanol two-bottle choice tasks. The system measures preference over time and changes in bout microstructure, with undisturbed recordings tested up to 7 d. All designs and software are open-source to allow other researchers to build on the system and adapt LIQ HD to their animal home cages.

## Significance Statement

Two-bottle choice drinking tasks are traditionally performed by periodically weighing bottles, which is cumbersome and lacks temporal resolution. Several open-source tools have been developed to improve drink monitoring in various settings. However, no open-source devices have been designed specifically to investigate temporally precise two-bottle choice drinking behavior and bout microstructure during prolonged undisturbed tasks in mouse ventilated home cages. Our design, LIQ HD (Lick Instance Quantifier Home cage Device), is a home cage compatible system that utilizes capacitive sensors for highly accurate lick detection during two-bottle choice tasks in up to 18 cages driven by a single Arduino microcontroller. The system is low-cost, easy to build, and controlled via touchscreen with an intuitive graphical user interface.

## Introduction

Monitoring of fluid intake and drinking behaviors is a powerful toolset in neuroscience research. These data provide insight into maladaptive behaviors observed in a wide range of disorders, such as obesity, substance use, depression, and others. A rich literature describes key brain regions in which populations of neurons are drivers of thirst, anhedonia, circadian rhythms of fluid consumption, and drug and ethanol consumption. Typical examples of such studies use standard voluntary two-bottle choice tasks in which animals are provided bottles in the home cage, one containing water and the other an experimental solution. Measures of fluid intake and preference are then calculated from bottle weight measurements manually taken by experimenters.

While two-bottle choice tasks remain the most common method for studying voluntary intake in rodents, performing the task manually is cumbersome, as data are traditionally collected by taking weight measurements throughout a specific time period, usually 1–3 d. Although this technique provides valuable information to researchers, it lacks temporal resolution. Increasing the frequency of bottle weighing increases variability and adds additional stress, as the animals must be disturbed to collect the data. Commercially available systems can track drinking behavior in a more automatized fashion in a home cage environment (Mingrone et al., 2020). These automated home cage monitoring systems are valuable because they generate data from a substantial number of different behavioral and metabolic measurements and drinking behavior. However, they are costly, require trained personnel, limit the number of cages that can be used, and are only available at a limited number of research institutions. Similarly, operant conditioning chambers can be used for assaying motivated behaviors related to fluid intake and tracking fine details associated with these behaviors, including lick microstructures; however, these tasks are not performed in a home cage environment and similarly require additional equipment and specialized training.

Caveats like those described above have inspired groups to develop open-source tools to study rodent drinking behaviors. The technology to detect licks (“lickometer”) or drink events for these devices has generally fallen into three categories: electrical lick sensors, optical lick sensors, and force lick sensors (Weijnen, 1998; Ulman et al., 2008). Early lickometer designs, although accurate, were hindered by a lack of easy-to-use commercially available components (Mundl and Malmo, 1979; Dole et al., 1983) or suboptimal compatibility with the home cage environment (Schoenbaum et al., 2001). Newer systems typically involve the use of inexpensive, commercially available sensors and components controlled by either a standard computer or microcontrollers, such as an Arduino or Raspberry Pi. Recently, two versions of infrared (IR) photobeam-break sensor based systems have been a common choice for detecting rodent drink events in a home cage environment (Frie and Khokhar, 2019; Godynyuk et al., 2019; Slivicki et al., 2023). The devices generated by these groups have filled many user needs, chiefly generating easy-to-use open-source designs that greatly improved the temporal resolution of drinking data. Godynyuk et al. (2019) created a mouse system optimized for use with *in vivo* recordings, such as fiber photometry, and Frie and Khokhar (2019) built off this first system by adapting it for rat cages and adding an innovative capacitance-sensing eTape to monitor changes in fluid levels within each water bottle. However, as IR sensors may be triggered by the animal’s snout in addition to its tongue, they detect drinking events but lack the ability of detect individual licks.

The use of electrical-sensing or capacitive-sensing has shown to have superior accuracy in detecting licks (Parkison et al., 2012; Longley et al., 2017; Melo et al., 2022). However, previously developed electrical-sensing devices are not designed to be compatible with a home cage because they require the use of a metal floor plate in a custom-built enclosure (Raymond et al., 2018; Melo et al., 2022). Devices that use a capacitive sensor on a chip do not require a metal plate, providing greater design flexibility. Capacitive sensors have allowed groups to design systems optimized for detecting licks in combination with recording movement (Parkison et al., 2012) and rat home cage operant devices (Longley et al., 2017). Thus, our goal was to design a device that utilizes capacitive sensing on a chip for accuracy and consistency, is compatible with a proper undisturbed mouse home cage environment and is able to record from multiple cages from a single microcontroller. In addition, we sought for the device to be used undisturbed for more extended periods of time by holding more fluid volume, have in-cage sensing components that are resistant to rodent destruction, and be intuitive and easy to build and use.

Here, we present LIQ HD (Lick Instance Quantifier Home cage Device): an affordable, intuitive, and easy-to-build device that utilizes capacitive sensor technology to track two-bottle choice drinking behavior in up to 18 rodent home cages, or 36 single bottles, on a minute-to-minute timescale running off a single Arduino microcontroller. The system is built with 3D-printed parts and affordable commercially available electronics. Further, our device is designed to be implemented directly in the animal’s home cage while on ventilated animal facility racks without any cage modification or requiring special housing conditions. The data for all cages are logged to a single SD card, allowing for efficient downstream analysis. Additionally, the system features a touchscreen controller with an intuitive graphical user interface to prevent the need for any code modification between experiments. Licks captured with LIQ HD strongly correlate with the volume consumed and has been tested in our hands with continued use over several months, with undisturbed runs for up to 7 d. Within the minute-by-minute data, in addition to lick number and lick duration, researchers can track drink preference as well as changes to the animals’ bout microstructure (bout duration, bout size, lick frequency, interlick interval) over time. It is our goal that LIQ HD will provide researchers with the tools necessary to gather fine-tuned drinking behavior data and streamline two-bottle choice paradigms, particularly those involving long-term home cage monitoring, such as ethanol two-bottle choice.

## Materials and Methods

### LIQ HD build instructions

**Figure 1.**
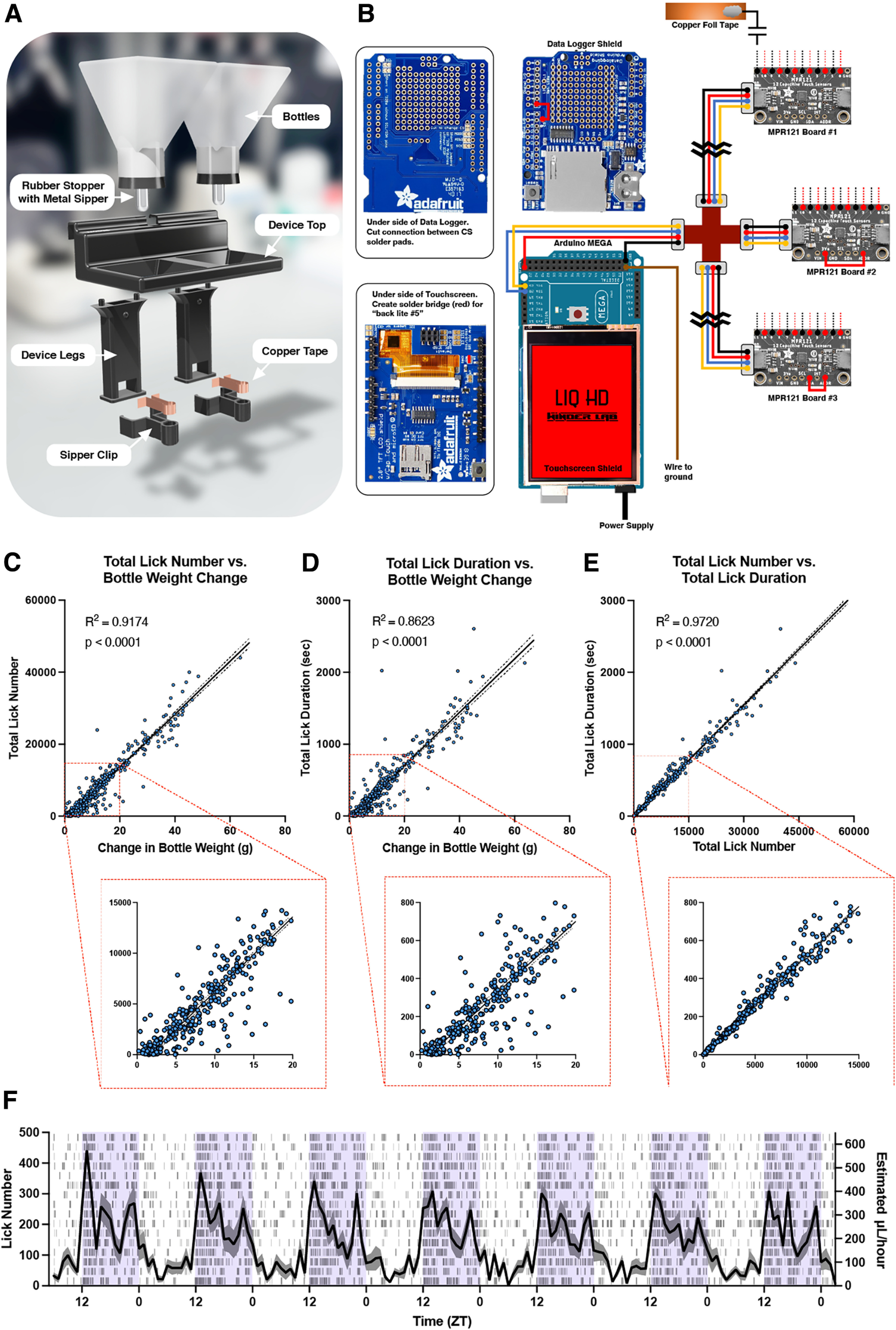
LIQ HD design and validation. ***A***, 3D rendering of LIQ HD disassembled components, including 3D-printed parts, rubber stoppers with sippers, and conductive copper foil tape. ***B***, LIQ HD electronic parts and wiring diagram. ***C***, Correlation between total lick number and change in bottle weight for each recording period. ***D***, Correlation between total lick duration and change in bottle weight for each recording period. ***E***, Correlation between total lick number and lick duration for each recording period. In correlation graphs, solid lines represent a fitted simple linear regression model, and dashed lines denote 95% confidence intervals. ***F***, Lick number and estimated water consumption throughout an undisturbed 7-d recording period with access to two water bottles. Data from both water bottles are combined to show the total intake per cage. The shaded purple area signifies the dark/active phase. The solid line represents the mean lick number and estimated intake in 1-h bins, and the shaded area represents ±SEM (*n* = 16 cages). The raster plot displays licks detected in 1-min bins for each cage. For the infrared photobeam-based two-bottle choice device design, wiring diagram, and validation, please see Extended Data Figure 1-1.

Materials for the build are listed in Table 1. LIQ HD is built from commercially available electronic components combined with 3D-printed parts. The system controls three separate 12-channel MPR121 capacitive sensor breakout boards (Adafruit) for a total of 18 individual devices (with two sippers each) and runs off a single Arduino Mega microcontroller (Fig. 1*A*,*B*). This capacitance-based sensor determines touch events by calculating a change in capacitance, or the ability of an object or substance to store an electric charge. This method does not require for a circuit to be completed for touch detection, thus there is no need for a conductive plate in the animal cage, and normal bedding can be used. Each metal sipper is in contact with conductive copper foil tape that is wired to an MPR121 capacitance sensor breakout board. With only the very tip of the sipper exposed, the device can sense individual licks without interference from other parts of the animal. While we have not done so, the Arduino code can be modified to support up to four separate MPR121 boards for a total of 24 devices (48 sippers). Date and time are kept with the Adafruit Data Logger Shield, which keeps time even if the device is unplugged or reset. The Data Logger Shield also writes the data to a single file on an SD card for efficient downstream analysis. Finally, a 2.8” Adafruit Touchscreen Shield is used to display an easy-to-use graphical user interface. This allows for the user to change device settings (time, light/dark schedule, sensor sensitivity, metrics to record), to mount/eject the SD card, and to start/stop/pause recordings. The screen also displays the total number of licks for each sipper during recordings (which can be refreshed to the user’s convenience) and will prompt the user if there are any errors, such as disconnected sensors or SD card.

10.1523/ENEURO.0506-22.2023.f1-1 Extended Data Figure 1-1 Infrared photobeam-based two-bottle choice device design and validation. ***A***, Electronic parts and wiring diagram for the beam-break system. ***B***, 3D rendering of beam-break device, including 3D-printed components, rubber stoppers and sippers, and photobeam sensors (red). ***C***, Correlation between total bout number and change in bottle weight for each recording period. ***D***, Correlation between total bout duration and change in bottle weight for each recording period. Solid lines represent fitted simple linear regression models, and dashed lines denote 95% confidence intervals. Download Figure 1-1, TIF file.

In building the electronics, the Data Logger Shield and Touchscreen Shield must be slightly modified to be made compatible with the Arduino Mega. First, install the Shield Stacking Headers on the Data Logger Shield. Then, solder a wire connecting the CS pin to pin 7. On the underside of the Data Logger Shield, cut the thin connection on the CS solder pad by carefully etching with a sharp blade. On the Touchscreen Shield, create a solder bridge across the pads labeled “back lite #5” to allow for dimming of the screen during the animals’ dark cycle. Mount the Data Logger Shield and the Touchscreen Shield onto the Arduino Mega by aligning and inserting the headers. The MPR121 breakout boards communicate with the Arduino Mega via I^2^C, which allows the microcontroller to communicate with multiple devices connected to the same pins if they have different I^2^C addresses. The MPR121 Board #1 will remain unmodified, but to modify the I^2^C address of the other MPR121 boards, solder a wire connection from “ADDR” to “3V” on one board (Board #2) and from “ADDR” to “SDA” on another (Board #3). Next, solder each pair of wires of the two-pin connectors to the sensor pins (0–11). For consistency, red wires are soldered to even numbers, and black to odd numbers. We provide additional reinforcement from accidental wire detachment by applying a layer of hot glue over the solder points. Inputs 0–11 on Board #1 correspond to sensors 1–12 (cages 1–6), inputs 0–11 on Board #2 correspond to sensors 13–24 (cages 7–12), and inputs 0–11 on Board #3 correspond to sensors 25–36 (cages 13–18). To connect the boards, attach the Qwiic cable with breadboard jumpers to the Arduino Mega (blue, pin 20; yellow, pin 21; red, 5V; black, GND) and secure with hot glue. Connect the four-way Qwiic Multiport Connector and plug in each MPR121 board with the Qwiic cables. Lastly, secure the device in the 3D-printed housing.

All 3D models were generated with Shapr3D and 3D-printed components were printed with PETG filament on an Ultimaker S5 printer. PETG was chosen for its high strength, durability, chemical resistance, ease of use, and food-safe properties. Bottles were printed with translucent filament and then coated internally with food-safe epoxy resin (meets regulation requirements for repeated use under United States Food and Drug Administration 21 CFR 177.2600) to prevent potential leaks and fill the space between printed layers. Fillings the spaces between the printed layers and creating a smooth inner surface also decreases the likelihood of bacterial and fungal growth. The 3D-printed material and resin are temperature sensitive and thus should not be used or cleaned with liquids >50°C. The bottles are washed by hand in warm water with high-quality soap (Dawn Platinum Dish Soap or Alconox Liquinox) or through a Steris Reliance 400XLS Laboratory Glassware Washer using a modified “Plastics” cycle (water temperature <50°C, no heated drying). If sterilization is required, the bottles can be sterilized via gas sterilization or UV sterilization methods. The in-cage device body was printed in pieces with black PETG. The legs were assembled to the upper portion with hot glue. For each device, two wire ends were soldered to the ends of two 3” × ¼” pieces of conductive copper tape. Copper tape is adhered to the inner part of each sipper clip, and wires are threaded up through the device body and out of the top. For consistency, red wire was used for the left side and black wire for the right side. Secure the sipper clips to the device legs with hot glue. Finally, solder the other two-pin connectors to the device wires for easy connection to the MPR121 inputs.

The LIQ HD Arduino code was uploaded using the open-source Arduino IDE software (version 1.8.14 on MacOS). Users must first install the necessary libraries through the Arduino IDE before uploading the code. A detailed step-by-step guide, along with the Arduino code and 3D models, can be found at https://github.com/nickpetersen93/LIQ_HD.

### Operation instructions

The device starts up with a splash screen followed by the device home page. The home page displays the date, time, and various buttons. Press the cogwheel icon to access the settings page, where the user can modify the date, time, light/dark cycle times, sensor sensitivity and auto calibration settings, parameters to record, bin size, and SD sync interval. Default settings are preloaded, but users should determine which sensor threshold works best for them. On the home page, the user can designate which side the “experimental” solution is on in the cages (i.e., sucrose, quinine, ethanol, etc.) before pressing “Start” to initiate recording. The SD card can also be mounted and ejected to allow for users to transfer data.

To begin recording, first ensure all devices are secured in the animal cages with the sippers properly placed in the clips. Connect each two-pin wire connector before initiating the recording with the “Start” button. After “Start” is pressed, the screen will display the data file name for 2 s and the sensors will calibrate. It is vital that the animals are not actively drinking and that the user steps away from the device during calibration for accurate measurements. Data are logged in 1-min bins and saved to the SD card every 10 min by default. On the recording page, the screen will display the cumulative lick number for the sippers in each cage. While these values are updated internally every minute, the user must press “Refresh” to display the updated values. The user also has the option to pause the recording with the “Pause” button. Pausing the recording prevents any new licks from being recorded, safely ejects the SD card for data transfer, and writes a line in the data spreadsheet indicating that the recording was paused. Upon pressing “Resume,” the SD card is mounted, the data file name will be displayed for 2 s, and the sensors will recalibrate. If the SD card fails or is removed at any point during the recording, the screen will display a warning along with the date and time that the recording failed. Rarely the I^2^C communication on the Arduino can lock up because of glitches or electrical interference. We have included a timeout detection in the code to identify lock-ups and resume recording without losing significant time. In this case, sensors will be restarted and recalibrated automatically. A line in the data will be logged if a timeout occurs. When the user has finished recording, pressing “Save & Quit” will sync any unsaved data to the SD card and return the device to the home page.

### Infrared photobeam-break device build

The IR photobeam-based drink monitoring device was built from 3D-printed parts and commercially available sensors and components. The design of the device was based on designs from Frie and Khokhar (2019) and Godynyuk et al. (2019) with modifications to allow for 16 cages to be recorded from a single Arduino Mega (Extended Data Fig. 1-1*A*). Briefly, 3D-printed parts were printed and assembled as described above. Beam-break sensor boards were assembled as previously described (Frie and Khokhar, 2019; Godynyuk et al., 2019) and secured into the 3D-printed device. A total of 32 photobeam sensors were wired to the Arduino input/output pins. A 1.3” OLED screen and two buttons were included to display total drink bout numbers for each cage and to operate the device. For this device, bout number is defined as the number of times the photobeam was interrupted and bout duration is the amount of time the beam was broken. Data were collected in 1-min increments.

### Determination of drinking bouts with LIQ HD

Lick number is defined as the number of times the animal licked the sipper, while lick duration is defined as the actual contact time on the sipper. As previously described (Siciliano et al., 2019), the start of a drinking bout is defined as three licks in <1 s and the end of a drinking bout is defined as no licks within 3 s. Bout duration is defined as the bout time minus the 3-s deadtime at the end of each bout. Bout size is defined as the number of licks that occurred during each bout. The bout lick number is defined as the number of licks that occur only during bouts, and the bout lick duration is the sipper contact time only during bouts. Lick frequency, defined as licks per second during bouts, for each bin is calculated by dividing the total bout length by the total bout duration in seconds:

$$Lick\;Frequency\left(Hz\right)=\frac{Bout\;Length\;(licks)}{Bout\;Duration\;(sec)}.$$

The estimated interlick interval is defined as the time between the offset of a lick and the onset of the subsequent lick. The average interlick interval for each bin is calculated by subtracting the total bout duration in milliseconds by the total bout lick duration and dividing by the total bout length:

$$Estimated\;Inter-Lick\;Interval\;\left(ms\right)=\frac{Bout\;Duration\;(ms)-Bout\;Lick\;Duration\;(ms)}{Bout\;Length\;(licks)}.$$

Bout microstructure did not significantly differ between the light and dark phase (Extended Data Fig. 4-1); thus, bout analysis is binned in 24-h bins. Occasionally, the touch sensors do not release until touched again, which can erroneously inflate lick duration counts and be displayed in the data as bins with a lick duration value but without any recorded licks. For 1-min bins that have a lick duration value without a lick number value, or bins where the average lick duration (lick duration/lick number) was over 300 ms, lick duration was changed to 0.

### Animals

Female C57BL/6J mice (eight weeks of age) were purchased from The Jackson Laboratory (#000664). Female C57BL/6J mice were used because of their established use in voluntary ethanol drinking paradigms, where female C57BL/6J mice drink significantly more ethanol and form a stronger preference for ethanol over water compared with male C57BL/6J mice (Middaugh et al., 1999; Hwa et al., 2011; Centanni et al., 2019; Sneddon et al., 2019). Mice were allowed to habituate to the animal facility for at least 7 d before the start of experimentation. All mice were singly housed in Lab Products Super Mouse 750 Ventilated Cages on a standard 12/12 h light/dark cycle at 22–25°C with food and water available *ad libitum*. All fluid measurements conducted by experimenters took place during the light phase. All experiments were approved by the Vanderbilt University Institutional Animal Care and Use Committee (IACUC) and were conducted in accordance with the guidelines set in the *Guide for the Care and Use of Laboratory Animals* of the National Institutes of Health.

### Two-bottle voluntary choice tasks

For each experiment, mice were singly housed in cages containing LIQ devices. Each device held two bottles. Every experiment began with a 7-d habituation period, during which both bottles contained only water, to allow the mice to acclimate to the housing conditions and the presence of two bottles. During this time no measurements were taken. Next, weights were taken every 48–72 h (on Monday, Wednesday, and Friday) for experiment 1 or every 7 d (every Friday) for experiment 2 to gain baseline fluid intake levels from both bottles containing only water. Bottle placement was swapped each time bottles were weighed to account for potential side biases. Following 7 d of water-only measurements, the fluid in one of the bottles was changed to either sucrose, quinine, or ethanol (as described below) while the second bottle remained filled with water. Again, for experiment 1, weights were taken by experimenters every 48–72 h (on Monday, Wednesday, and Friday), and for experiment 2, weights were taken every 7 d (every Friday). As before, bottle placement was swapped every time bottles were weighed. For each experimental solution, a dose–response curve was performed in order from lowest to highest dose. For experiment 1, sucrose and quinine doses were each provided for 48–72 h (72 h for 0.5% sucrose and 48 h each for 1% and 10% sucrose) before being switched to the next concentration. For experiment 2, 3% and 7% ethanol solutions were each provided for one week, followed by four weeks of 10% ethanol. The following doses were selected based on previous studies indicating altered intake and/or preference across a dose–response curve:

Sucrose: 0.5%, 1.0%, and 10% sucrose in tap water (Zukerman et al., 2009; Glendinning et al., 2010; Doyle et al., 2021)

Quinine: 0.01, 0.03, and 0.1 g/l in tap water (Hodge et al., 1999; Winters et al., 2021)

Ethanol: 3%, 7%, and 10% ethanol in tap water (Hodge et al., 1999; Centanni et al., 2019; Winters et al., 2021)

### Statistical analysis

Data were first extracted and processed with a custom MATLAB script. Unless otherwise stated, data were binned into 1-h bins. For the calculation of bottle preference over time, the binned data were smoothed with a moving average with a sliding window length of 6. Statistical analyses were performed with Prism 9 (GraphPad). Pearson correlation coefficients and simple linear regressions were computed for all correlation pairs. Repeated measures ANOVA with corrections for multiple comparisons were performed as indicated in the figure legends.

### Code accessibility

The code/software described in the paper is freely available online at https://github.com/nickpetersen93/LIQ_HD. The code is available as Extended Data 1.

10.1523/ENEURO.0506-22.2023.ed1 Extended Data 1 LIQ HD Arduino source code used in this study. Download Extended Data 1, ZIP file.

## Results

### Assessment of modified beam-break two-bottle choice system

The initial two-bottle choice pilot experiment conducted in the lab used a modified infrared (IR) beam break system (Frie and Khokhar, 2019; Godynyuk et al., 2019; Extended Data Fig. 1-1*A*) and singly housed female C57BL/6J mice. Specifically, the design of our device was adapted to expand the volume the bottles could hold to 90 ml each and record 16 cages from a single Arduino microcontroller. Following a week of habituating to two bottles containing water, four additional days of water-only availability were measured by the experimenter in daily sessions. This period was followed by a solution series, during which the fluid in one of the bottles was replaced with the following solutions: two consecutive days of 1% sucrose, 1 d of 10% sucrose, and 1 d of 0.1 g/l quinine. During this series, fluid intake was measured daily by an experimenter. To assess accuracy of the beam break-based measurements, the daily weight change values determined by experimenter intervention were correlated with the IR beam-break number and duration of bream-breaks recorded during the same recording period. When the data across the water and solution series were compiled, we were able to closely replicate previous findings (Godynyuk et al., 2019; Extended Data Fig. 1-1*B*,*C*). The correlation between total beam-break bout number and change in bottle weight (*R*^2^ = 0.3810, *F*_(1,85)_ = 52.31, *p* < 0.0001) as well as the correlation between total beam-break bout duration (seconds) and change in bottle weight (*R*^2^ = 0.3844, *F*_(1,85)_ = 53.07, *p* < 0.0001), were statistically significant. Unfortunately, we encountered several issues in our redesign throughout the recording period. First, multiple devices required repair or replacement because of mice chewing and damaging the photobeam sensors. Second, because of our selected sipper type and the placement over the sensor, we found that drips hanging from the bottom of the sippers would trigger the sensor and overcount bout duration. Further, mice frequently attempted to bury the sensors with bedding, especially in the initial recording days, which also erroneously triggered the sensors. In the presented data, data points were removed in bins where the bout duration lasted an entire bin or where there was a recorded bout duration without a corresponding bout number.

### Assessment of LIQ HD two-bottle choice system

While the data collected with the IR beam-break device significantly correlated with change in bottle weight and were nearly identical to published results, we sought to further improve on the accuracy and reliability of the behavioral recordings as well as increase the precision of the recorded data by designing a device, LIQ HD, that utilizes capacitive sensing technology to detect single licks. We took the modified design described above and replaced the IR beam-break sensors with a capacitance-sensing system. To validate the ability of LIQ HD to measure intake behaviors accurately, we performed a new series of two-bottle choice experiments with singly housed female C57BL/6J mice. Bottle weights were measured every 2–3 d (experiment 1) or every 7 d (experiment 2). Experiment 1 consisted of two groups of mice (8 mice each), where one group received a sucrose dose–response (0.5%, 1%, and 10% sucrose vs water) and the other group received a quinine dose response (0.01, 0.03, and 0.1 g/l quinine vs water). In experiment 2, 16 mice went through a water-only two-bottle choice session, followed by 8 of those mice advancing through an additional ethanol dose–response paradigm (3%, 7%, and 10% EtOH vs water). In both experiments, mice were first habituated to the LIQ devices with water in both bottles for one week, where no measurements were taken, and then given an additional week of water only. For experiment 1, the experimental solutions (sucrose and quinine) were changed every 2–3 d, which coincided with weight measurements and swapping the sides of the bottles. For experiment 2, the ethanol and water bottles were weighed, changed, and swapped sides every 7 d. The LIQ HD and bottle measurement data from experiments 1 and 2 were combined to correlate the total lick numbers and total lick durations from each recording period with the corresponding bottle weight measurements (Fig. 1*C*,*D*). We observed a strong, significant correlation between both total lick number and change in bottle weight (*R*^2^ = 0.9174, *F*_(1,363)_ = 4034, *p* < 0.0001) as well as total lick duration and change in bottle weight (*R*^2^ = 0.8623, *F*_(1,363)_ = 2273, *p* < 0.0001), substantially higher *R*^2^ values than our previous IR beam-break based system (Extended Data Fig. 1-1). The fitted regression model for the correlation between lick number and change in bottle weight is Y = 736.4*X − 1172 (slope 95% CI, 713.6–759.2). Thus, on average, the mice took 736 licks to drink 1 ml of fluid, or 1.4 μL per lick. As expected, total lick number and total lick duration also strongly correlate (*R*^2^ = 0.9720, *F*_(1,363)_ = 12 601, *p* < 0.0001) with a fitted regression model of Y = 0.05,132*X + 8.027 (slope 95% CI, 0.050–0.052; Fig. 1*E*). On average, the individual lick duration was 51 ms. These metrics are consistent with the literature (Mundl and Malmo, 1979; Parkison et al., 2012; Rossi and Yin, 2015; Bollu et al., 2021), providing further evidence that LIQ HD is detecting individual lick events with high fidelity. In our tests, the LIQ HD system ran reliably undisturbed between periods of bottle weight measurements for at least 7 d (Fig. 1*F*).

### Experiment 1, LIQ HD validation in sucrose and quinine dose–response two-bottle choice tasks

**Figure 2.**
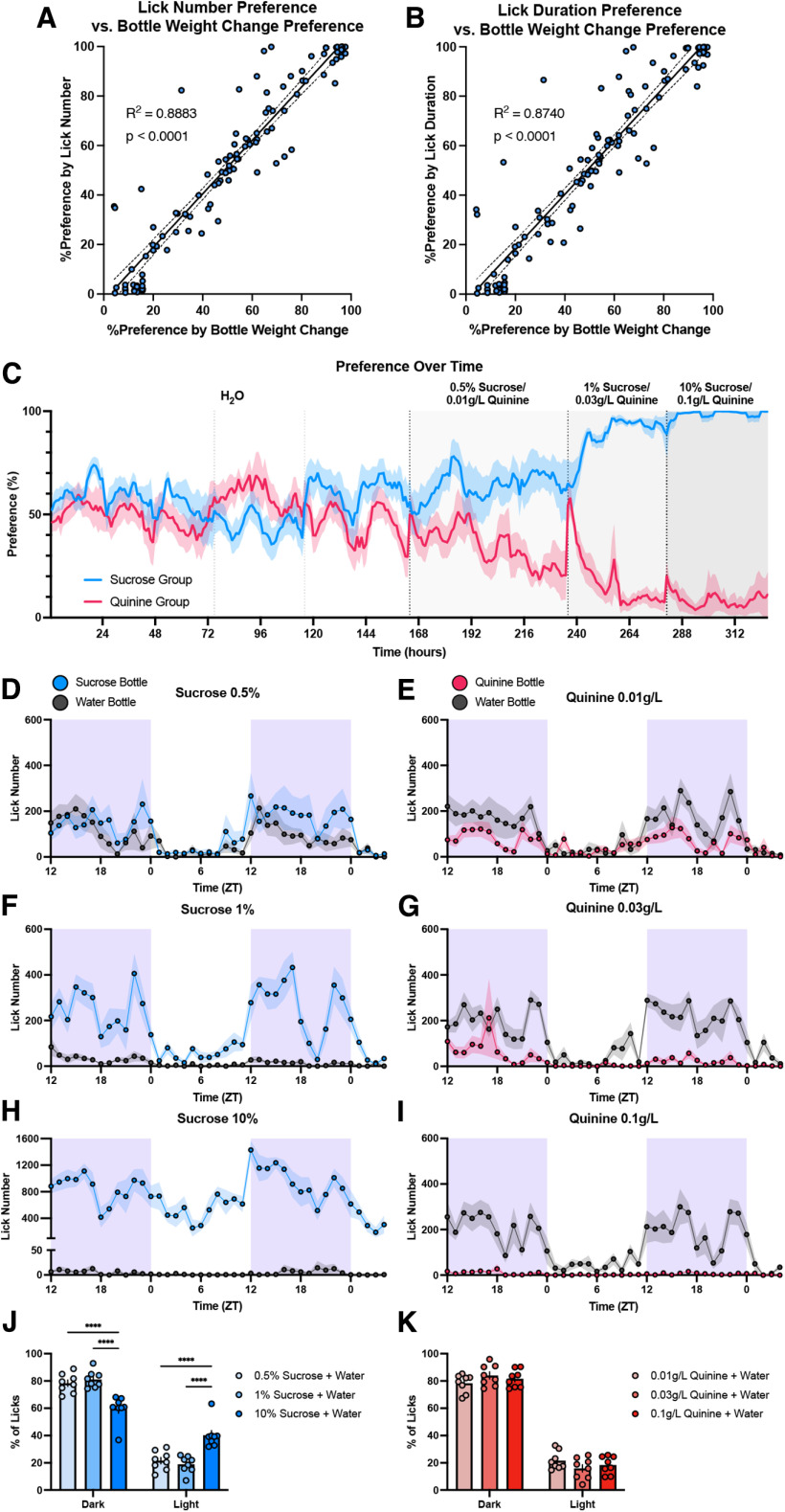
Using LIQ HD to investigate changes in drink preference and light/dark cycle drinking patterns in sucrose and quinine two-bottle choice paradigms. ***A***, Correlation between percent preference calculated with lick number and percent preference calculated with change in bottle weight for each recording period. ***B***, Correlation between percent preference calculated with lick duration and percent preference calculated with change in bottle weight for each recording period. Solid lines represent fitted simple linear regression models, and dashed lines denote 95% confidence intervals. ***C***, Preference over time in 1-h bins for the sucrose and quinine two-bottle choice dose–response paradigm. The solid line represents smoothed mean (sliding window length of 6), and the shaded area signifies ±SEM (*n* = 8 cages for each group). Vertical dashed lines indicate when bottles swapped sides and when indicated, a change in experimental solution in one bottle. ***D–I***, Lick number over time in 1-h bins for recording periods for the two-bottle choice sucrose and quinine dose response paradigms. The shaded purple area signifies the dark/active phase. ***J***, Percentage of total licks that occur during the dark and light phases for recording periods of the sucrose dose–response paradigm (repeated measures two-way ANOVA, with the Geisser–Greenhouse correction and Tukey’s multiple comparisons test). During access to 10% sucrose, the percentage of licks that occurred during the dark phase was significantly decreased, and the percentage of licks that occurred during the light phase was significantly increased when compared with 0.5% and 1% sucrose availability. ***K***, Percentage of total licks during the dark and light phases for the quinine dose–response paradigm (repeated measures two-way ANOVA, with the Geisser–Greenhouse correction and Tukey’s multiple comparisons test). Shaded areas and error bars represent ±SEM; ****p < 0.0001.

To test the LIQ HD system in common two-bottle choice paradigms, female C57BL/6J mice were split into two groups. One group underwent a two-bottle choice paradigm with a sucrose dose–response curve, and the other a quinine dose–response curve. Bottles were weighed and swapped sides every 2–3 d. When the recorded data were aggregated, we observed a strong, significant correlation between preference score calculated by total lick number and preference score calculated by bottle weight change (*R*^2^ = 0.8883, *F*_(1,110)_ = 874.5, *p* < 0.0001) as well as between preference score calculated by total lick duration and preference score calculated bottle weight change (*R*^2^ = 0.8740, *F*_(1,110)_ = 763, *p* < 0.0001; Fig. 2*A*,*B*). Additionally, with the added temporal resolution of LIQ HD over standard experimenter-determined measures, we also investigated changes in drink preference over time (Fig. 2*C*). As expected, preference for the sucrose bottle over water increased with increasing concentrations of sucrose, and preference for the quinine bottle over water decreased with increasing concentrations of quinine (Fig. 2*C*). Moreover, we examined changes in drinking behaviors over the light and dark cycle to detect potential deviations from the typical circadian drinking patterns, with examples displayed in Figure 2*D–I*. Overall, for the percent of total licks occurring per cycle in the sucrose group there was a significant main effect of light cycle (*F*_(1,21)_ = 209.7, *p* < 0.0001) and interaction of light cycle × sucrose concentration (*F*_(2,21)_ = 17.64, *p* < 0.0001). While the mice increased their total licks at the sucrose bottle as the sucrose concentration was increased from 0.5% to 1% sucrose, the percentage of the overall licks that occurred during the light and dark phases did not change (Fig. 2*J*). In addition to further increasing total licks at the sucrose bottle during access to 10% sucrose (Fig. 2*H*), the mice also displayed a unique increase in percent of sucrose consumption occurring during the light phase (0.5% vs 10% sucrose, *p* < 0.0001, 1% vs 10% sucrose, *p* < 0.0001), and corresponding decrease in the dark phase (0.5% vs 10% sucrose, *p* < 0.0001, 1% vs 10% sucrose, *p* < 0.0001; Fig. 2*J*). Mice exposed to increasing concentrations of quinine with water did not display any changes in the light cycle drinking pattern (Fig. 2*K*; significant main effect of light cycle only, *F*_(1,21)_ = 485.5, *p* < 0.0001). Taken together, these data indicate that LIQ HD can accurately and consistently record overall preference scores, preference over time, and light cycle-dependent drinking patterns in classic two-bottle choice paradigms.

### Experiment 2, LIQ HD bout detection and microstructure analysis in a prolonged ethanol two-bottle choice task

Because LIQ HD was able to accurately quantify behavioral data from sucrose and quinine two-bottle choice tasks, we next sought to validate its use in longer duration tasks. To do this, we turned to a six-week continuous access ethanol two-bottle choice task. Further, given that LIQ HD can detect individual lick events, we sought to determine whether the system is able to detect drinking bouts and record bout microstructure. To do this, we coded bout detection directly to the main LIQ HD Arduino code, where a “bout” begins when an animal licks at least three times within 1 s and ends when no licks have occurred over 3 s (Siciliano et al., 2019). With this we can also determine the lick number and lick duration during bouts, which allows us to calculate lick frequency and an estimated interlick interval.

**Figure 3.**
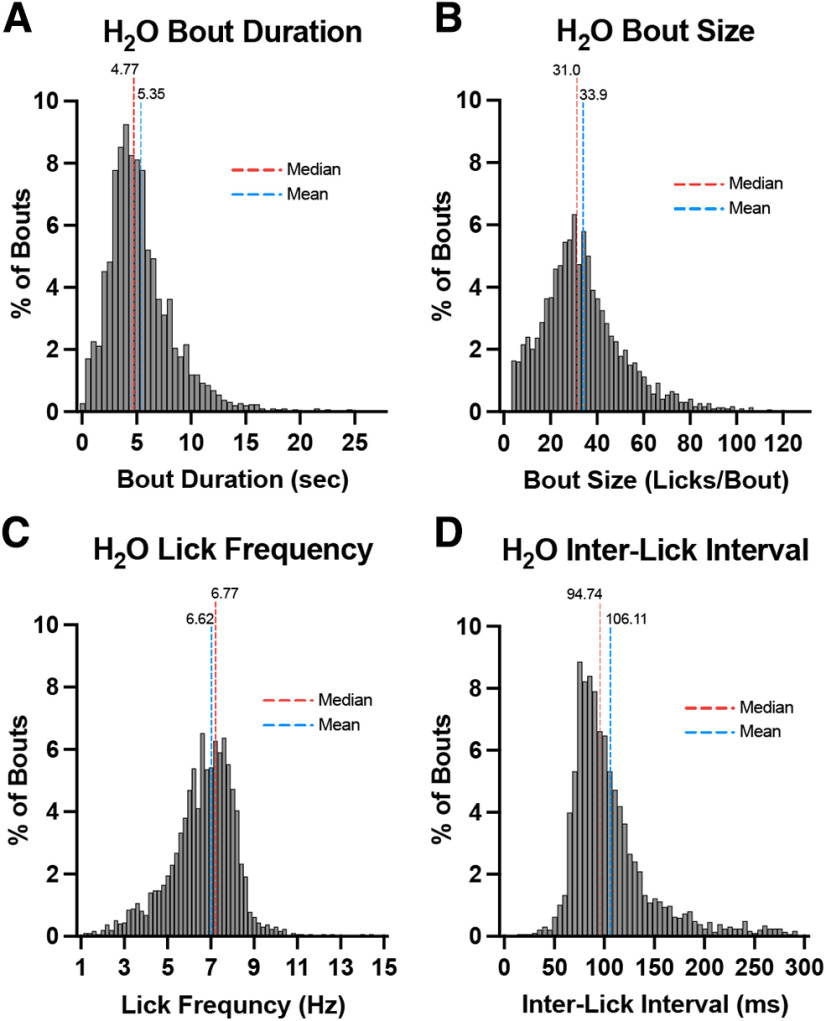
Histograms of average baseline bout microstructure measurements from 1-h bins during one-week access to two water bottles (*N* = 16 mice, *n* = 32 bottles). Histogram displaying bout duration (***A***), bout size (***B***), lick frequency (***C***), and estimated interlick interval (***D***). Red and blue dashed lines represent the median and mean, respectively.

First, to test the LIQ HD bout detection, 16 female C57BL/6J mice underwent a two-bottle choice task with access to two water bottles. Mice were first habituated for one week with a LIQ device with water in both bottles, during which no measurements were taken. Water-related measurements were then taken during a subsequent week of water-only access. We also sought to determine whether LIQ HD is capable to run for prolonged undisturbed recording periods, thus experimenter measurements of bottle weights were taken only every 7 d. The LIQ data from each sipper (16 cages, 32 sippers) was binned into 1-h bins and the average individual bout duration, bout size, bout lick frequency, and bout estimated interlick interval were calculated. Estimated interlick interval values >300ms were excluded from the analysis. The mean and median individual bout duration (seconds) during the water drinking period (*n* = 2919) were 5.35 ± 0.06 (SEM) and 4.77 (IQR 3.34–6.67; Fig. 3*A*). The mean and median of individual bout size (licks per bout) during the water drinking period (*n* = 2914) were 33.9 ± 0.34 (SEM) and 31.0 (IQR 22.0–42.5; Fig. 3*B*). The mean and median of lick frequency (Hz; *n* = 2915) were 6.62 ± 0.03 (SEM) and 6.77 (IQR 5.81–7.63; Fig. 3*C*). The mean and median of estimated interlick interval (milliseconds) during the water drinking period (*n* = 2858) were 106 ± 0.79 (SEM) and 94.7 (IQR 79.1–118; Fig. 3*D*). These findings are consistent with results from previous studies (Boughter et al., 2007; Johnson et al., 2010; Parkison et al., 2012; Raymond et al., 2018; Bollu et al., 2021).

Following the LIQ bout detection validation, eight of the female C57BL/6J mice underwent a two-bottle choice ethanol drinking paradigm. The two-bottle choice paradigm consisted of an ethanol ramp where one of the water bottles was replaced with an ethanol solution. During the ramp, mice received one week of 3% ethanol, one week of 7% ethanol, and four weeks of 10% ethanol. As stated above, the system can accurately and consistently record drinking behavior over a 7-d period (Fig. 1*F*). Moreover, LIQ HD withstood eight weeks (including water only access and habituation) of continuous usage without any devices needing repair or replacement during experimentation.

**Figure 4.**
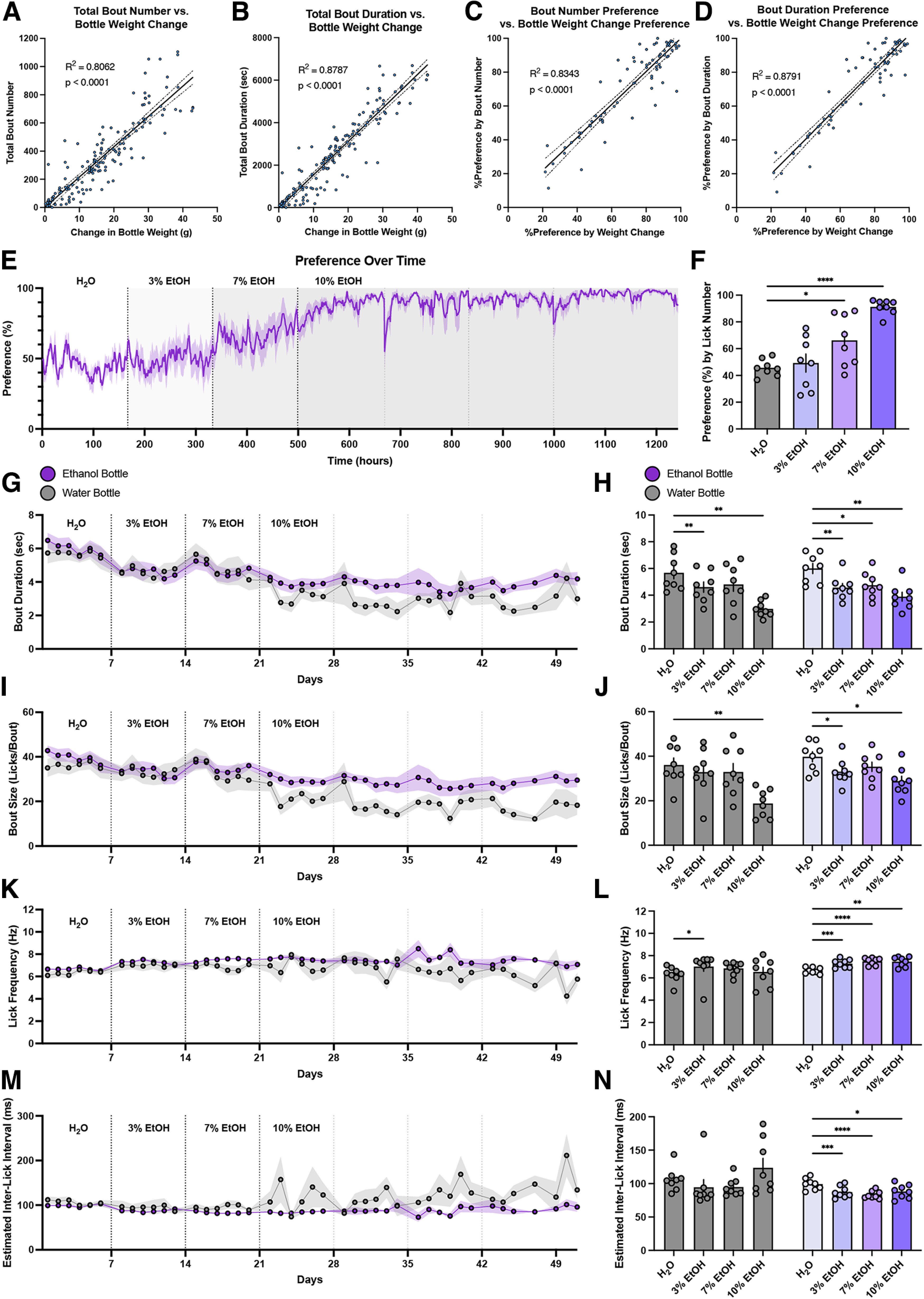
Validation of LIQ HD bout detection and bout microstructure in an ethanol two-bottle choice paradigm. ***A***, Correlation between total bout number and change in bottle weight for each recording period. ***B***, Correlation between total bout duration and change in bottle weight for each recording period. ***C***, Correlation between percent preference calculated with bout number and percent preference calculated with change in bottle weight for each recording period. ***D***, Correlation between percent preference calculated with bout duration and percent preference calculated with change in bottle weight for each recording period. Solid lines represent a fitted simple linear regression model, and dashed lines denote 95% confidence intervals. ***E***, Preference over time in 1-h bins for the ethanol two-bottle choice paradigm. The solid line represents smoothed mean (sliding window length of 6), and shaded area signifies ±SEM (*n* = 8 cages). Vertical dashed lines indicate when bottles swapped sides and when indicated, a change of experimental solution in one bottle. ***F***, Average preference for experimental bottle during ethanol two-bottle choice paradigm (repeated measures one-way ANOVA, with the Geisser–Greenhouse correction and Dunnett’s multiple comparisons test). Mice show significantly increased preference compared with baseline for 7% and 10% ethanol. Bout duration (***G***), bout size (***I***), lick frequency (***K***), and estimated interlick interval (***M***) over time in 24-h bins for water and ethanol bottles. Bout microstructure data were grouped into 24-h bins because there were no significant differences between the light and dark cycle (Extended Data Fig. 4-1). Average bout duration (***H***), bout size (***J***), lick frequency (***L***), and estimated interlick interval (***N***) during access to two water bottles and access to water with increasing concentrations of ethanol (repeated measures two-way ANOVA, with the Geisser–Greenhouse correction and Dunnett’s multiple comparisons test). At the ethanol bottle, mice display a significant decrease in bout duration (***G***), bout size (***I***), and interlick interval (***N***) with a significant increase in lick frequency (***L***) during access to increasing concentrations of ethanol. At the water bottle, mice display a significant increase in lick frequency only during access to 3% ethanol (***L***), a significant decrease in bout duration during access to 3% and 10% ethanol (***H***), and a significant decrease in bout size only during access to 10% ethanol (***J***). Shaded areas and error bars represent ±SEM; *p < 0.05, **p < 0.01, ***p < 0.001, ****p < 0.0001.

To determine the correlation of bout number and bout duration with change in bottle weight, as well as the correlation of preference score calculated by bout number and bout duration with the preference score calculated by change in bottle weight, the water two-bottle choice and ethanol two-bottle choice data were aggregated. We observed a strong, significant correlation between total bout number and change in bottle weight (*R*^2^ = 0.8062, *F*_(1,156)_ = 648.8, *p* < 0.0001) as well as between total bout duration and change in bottle weight (*R*^2^ = 0.8787, *F*_(1,156)_ = 1130, *p* < 0.0001; Fig. 4*A*,*B*). The fitted regression models for bout number versus change in bottle weight and bout duration versus change in bottle weight are Y = 21.51*X + 0.8541 (slope 95% CI, 19.84–23.17) and Y = 157.533*X − 37 270 (slope 95% CI, 148.276–166.790), respectively. These data indicate that on average mice drink 1 ml over 21.5 bouts, or 46.5 μl per bout, and on average take 157.5 s to drink 1 ml during bouts, or 6.35 μl/s. We also found a strong, significant correlation between preference score calculated by total bout number and preference score calculated by bottle weight change (*R*^2^ = 0.8343, *F*_(1,69)_ = 347.4, *p* < 0.0001) as well as between preference score calculated by total bout duration and preference score calculated by bottle weight change (*R*^2^ = 0.8791, *F*_(1,69)_ = 501.8, *p* < 0.0001; Fig. 4*C*,*D*). While bout number and bout duration may not be as reliable of a predictor of total change in bottle weight compared with total lick number (*R*^2^ = 0.9174), these data suggest that the LIQ HD bout detection software can accurately detect individual clustered drinking events throughout long recording periods.

10.1523/ENEURO.0506-22.2023.f4-1 Extended Data Figure 4-1 Bout microstructure does not significantly differ between the light and dark cycle. A total of 16 mice were given access to two water bottles for a one-week recording period. The data for the 32 bottles were pooled and binned into 12-h bins to determine differences in bout microstructure between the light and dark cycle. Mixed-effects models with the Geisser–Greenhouse correction using a compound symmetry covariance matrix and fit using restricted maximum likelihood (REML) revealed no significant main effect of light cycle for the average individual bout duration (*F*_(1,31)_ = 0.8731, *p* = 0.3573), average individual bout size (*F*_(1,31)_ = 0.2621, *p* = 0.6123), average bout lick frequency (*F*_(1,31)_ = 1.019, *p* = 0.3206), or average ILI (*F*_(1,31)_ = 0.1218, *p* = 0.7294; *N* = 16 mice, *n* = 32 bottles). Download Figure 4-1, TIF file.

In addition to preference over time in the prolonged ethanol two-bottle choice paradigm, where mice showed expected significantly elevated preference for both 7% ethanol (*p* = 0.0481) and 10% ethanol (*p* < 0.0001) compared with water (Fig. 4*E*,*F*), LIQ HD can also be used to analyze drinking bout microstructure over time (Fig. 4*G–N*). Bout microstructure data were grouped into 24-h bins, as we did not observe differences between the light and dark cycle (Extended Data Fig. 4-1). Throughout this increasing preference for ethanol, the average bout duration, bout size, lick frequency and estimated interlick interval were altered. Although bouts at the ethanol bottle became more frequent compared with those at the water bottle, access to ethanol significantly decreased the average bout duration (significant main effect of ethanol concentration, *F*_(2.129,29.80)_ = 23.68, *p* < 0.0001) at both the water bottle (H_2_O vs 3% EtOH, *p* = 0.0096, H_2_O vs 10% EtOH, *p* = 0.0022) and the ethanol bottle (H_2_O vs 3% EtOH, *p* = 0.0055, H_2_O vs 7% EtOH, *p* = 0.0310, H_2_O vs 10% EtOH, *p* = 0.0054; Fig. 4*H*). Bout size also significantly decreased (significant main effect of ethanol concentration, *F*_(1.882,26.34)_ = 17.01, *p* < 0.0001) at the ethanol bottle during ethanol exposure (H_2_O vs 3% EtOH, *p* = 0.0288, H_2_O vs 10% EtOH, *p* = 0.0189), but only decreased at the water bottle during exposure to the highest ethanol dose (H_2_O vs 10% EtOH, *p* = 0.0047; Fig. 4*J*). Interestingly, lick frequency significantly increased (significant main effect of ethanol concentration, *F*_(2.300,32.20)_ = 8.236, *p* = 0.0008) at the ethanol bottle at all doses (H_2_O vs 3% EtOH, *p* = 0.0005, H_2_O vs 7% EtOH, *p* < 0.0001, H_2_O vs 10% EtOH, *p* = 0.0017; Fig. 4*L*) with a concurrent decrease in the estimated interlick interval (significant main effect of ethanol concentration, *F*_(2.300,32.20)_ = 8.236, *p* = 0.0008, significant interaction ethanol concentration × bottle *F*_(3,42)_ = 3.801, *p* = 0.0169, H_2_O vs 3% EtOH, *p* = 0.0002, H_2_O vs 7% EtOH, *p* < 0.0001, H_2_O vs 10% EtOH, *p* = 0.0236; Fig. 4*N*). We only observed an increase in lick frequency at the water bottle during access to 3% ethanol (H_2_O vs 3% EtOH, *p* = 0.0434) without any significant differences in estimated interlick interval (Fig. 4*L*,*N*). Overall, these data replicated known effects of prolonged, continuous access to increasing concentrations of ethanol in an undisturbed home cage environment on preference for ethanol over water and revealed significant changes to the bout lick microstructure in female C57BL/6J mice.

## Discussion

Here we present LIQ HD (Lick Instance Quantifier Home cage Device), a capacitance sensor-based two-bottle choice open-source system capable of detecting undisturbed licking behavior in a true rodent home cage environment. A single LIQ HD Arduino system records drinking behavior in up to 18 cages. The system includes a touchscreen with a graphical user interface for an intuitive user experience. LIQ HD detects single lick events, and lick number and duration strongly correlate with change in liquid volume as measured by manually weighing the bottles. The use of capacitive sensors significantly outperformed our modified beam-break sensor-based device (*R*^2^ of 0.9174 vs 0.3844). Additionally, each 3D-printed bottle holds roughly 90 ml of liquid, allowing for prolonged, undisturbed recording sessions. In this study we used LIQ HD continuously for several months with undisturbed recordings lasting up to 7 d. We observed that female C57BL/6J mice drank ∼7 ml of water per day, suggesting that the system could potentially run for multiple weeks undisturbed. It is important to note that the maximum length of the recording period will depend on preference values, mouse strain (Bachmanov et al., 2002), and the animal housing regulations of the research institution.

In a series of two-bottle choice paradigms, we show that LIQ HD accurately measures drink preference, and the minute-by-minute data also allow for the analysis of circadian drinking patterns. For example, here we show that access to 10% sucrose shifts the typical dark/light drinking pattern, with a significantly greater percentage of licks occurring in the light phase and less in the dark phase when compared with 0.5% or 1% sucrose availability (Fig. 2*J*). These features may be helpful in studying individual differences in drinking behaviors, such as investigating differences in the acquisition of drink preference or susceptibility to circadian dysregulation. Moreover, the ability of the LIQ HD to detect individual licks allows for analysis of the mouse bout microstructure, further simplified by our software which detects bouts directly from the Arduino source code. While it is challenging to compare lick microstructure across various experimental modalities, such as unlimited continuous access, intermittent access, operant conditioning tasks, etc., our baseline bout microstructure measurements are consistent with the data in the literature (Boughter et al., 2007; Johnson et al., 2010; Parkison et al., 2012; Raymond et al., 2018; Bollu et al., 2021). Using this bout detection and microstructure system, we observed significant changes to drinking structure over time in a classic ethanol two-bottle choice task. As previously reported, the female C57BL/6J mice significantly increase their preference for ethanol as the concentration increased from 3%, 7%, and 10% ethanol. With the increase in preference for ethanol, we found that the mice display a significant decrease in bout duration and bout size at the ethanol bottle, despite showing a strong increase in preference for the ethanol bottle (Fig. 4). The mice also show a significant increase in lick frequency with a concurrent decrease in the estimated interlick interval at the ethanol bottle. Bout size and lick rate, particularly the initial lick rate, are directly related to the palatability of the drinking solutions, making studying bout size a useful tool for understanding hedonic value in fluid consumption (Davis and Levine, 1977; Baird et al., 2005; Dwyer et al., 2011; Dwyer, 2012; Johnson, 2018). Interestingly, our data show opposing microstructure changes regarding this palpability interpretation, with ethanol decreasing bout size/duration but increasing lick frequency. This suggests that the effect of ethanol on bout microstructure is more complex than simply representing palpability. The reinforcing effects of chronic ethanol exposure may be counteracting the presumed decrease in palpability with increasing concentrations. In addition to investigation of hedonic stimuli, analysis of bout microstructure over time may serve as a useful tool in studying chronic manipulation of rhythmic central pattern generators of consummatory motor function (Brozek et al., 1996; Vajnerova and Brozek, 2002). Taken together, LIQ HD can be used to investigate drinking behavior and bout microstructure with high temporal resolution and accuracy for two-bottle choice tasks with prolonged undisturbed recording periods.

The use of capacitive touch sensors in the LIQ HD system has several advantages over our redesigned IR photobeam system. In addition to significantly improved accuracy, the capacitive sensors in LIQ HD are more resilient. In our experience, the photobeam sensors were frequently subject to destruction by rodent chewing. In the LIQ HD system, if the sipper remains in contact with the conductive copper foil tape, LIQ HD will continue to detect licks, creating a low likelihood that a mouse could destroy the device to the point that it malfunctions. During these experiments and initial pilot studies, the 16 LIQ HD devices ran for >100 d without any device failures. It is possible that if used with rats or animals with increased chewing behavior, such stress-exposed or opioid-exposed mice, the 3D-printed clips that secure the copper tape and sipper will require more frequent repair. However, we expect some damage and have accordingly designed the device so that the clip is easily removable and replaceable. Moreover, the capacitive sensor boards communicate with the Arduino controller though I^2^C communication rather than through the direct input/output pins on the microcontroller. This allows for many devices to run off a single Arduino, as the system is no longer limited to the number of available pins but rather the ability of the sensor boards to have unique I^2^C addresses. In its current state LIQ HD utilizes three 12-channel MPR121 capacitive sensor boards, for a total of 36 sippers, but it can be readily expanded by the end user to use four boards for 48 sippers. With the addition of an I^2^C address multiplexer, users can connect multiple sensor boards that have the same address, further scaling LIQ HD.

Like any system, LIQ HD has several limitations when considering its use. Unlike battery-powered systems, our system must be continuously plugged into an outlet and will stop recording if power is lost. The capacitive sensors may also be subject to electrical interference if not properly grounded. To avoid this interference, the listed power supply contains a grounding prong, and we recommend an additional grounding wire from the Arduino to another grounding source. In addition, the capacitive sensors likely render this system incompatible with electrophysiology, though we have not tested this. It is also important to note that because electrical wires increase capacitance and thus affect sensor sensitivity, it is essential that all wire lengths connecting cages are kept consistent and as short as possible. LIQ HD is also limited by the same factors that limit most two-bottle choice systems, such as requiring animals to be singly housed and requiring periodic switching of bottle sides to avoid side bias. Finally, our experiments are limited by the use of only female mice; however, previous work has shown that male and female C57Bl/6J mice do not have significant differences in drinking microstructure (Boughter et al., 2007), and we believe that any differences would not affect LIQ HD functionality. In fact, LIQ HD may be well suited to evaluate sex-dependent differences in changes to bout microstructure in future work.

To conclude, LIQ HD is an affordable, easy-to-build, multihome cage lickometer system that utilizes capacitive sensors for accurate lick detection. The devices hold sufficient liquid bottle weight change in two-bottles for prolonged undisturbed recordings of drinking behavior and bout microstructure and are highly resistant to functional damage from rodent manipulation. The current system is designed to record up to 18 cages simultaneously, and its precision can eliminate the need for cumbersome bottle weighing, thus rendering it suitable for high-throughput experiments. We encourage users to use the open-source code and designs to expand on our current system to make LIQ HD compatible with other home cages, increase the scale of recordings, and improve overall efficiency.

**Table 1:** Complete list of LIQ HD building components with manufacturer and distributor product information, quantity per system, price per component unit, and total cost in $USD

Item	Manufacturer (product #)	Distributor (product #)	Qty.	Price/unit ($USD)	Total ($USD)
Arduino Mega 2560 Rev3	Arduino (A000067)	Digi-Key (1050-1018-ND)	1	48.40	48.40
2.8” Capacitive Touch Shield	Adafruit (1947)	Digi-Key (1528-1050-ND)	1	44.95	44.95
Adafruit Data Logging Shield	Adafruit (1141)	Digi-Key (1528-1044-ND)	1	13.95	13.95
Shield Stacking Headers for Arduino	Adafruit (85)	Digi-Key (1528-1074-ND)	1	1.95	1.95
12.5 mm 3V CR1220 Lithium Battery	Jauch Quartz (CR 1220 JAUCH SB)	Digi-Key (1908- CR1220JAUCHSB-ND)	1	1.09	1.09
8GB microSD Card with Adapter	Adafruit (1294)	Adafruit (1294)	1	9.95	9.95
MPR121 12-Key Capacitive Touch Sensor Breakout	Adafruit (1982)	Digi-Key (1528-1038-ND)	3	7.95	23.85
12V 5A Switching Power Supply	Adafruit (352)	Digi-Key (1528-1664-ND)	1	24.95	24.95
Qwiic Multiport Connector	SparkFun (BOB-18012)	Digi-Key (1568-BOB-18012-ND)	1	2.10	2.10
Qwiic Cable Breadboard Jumper	SparkFun (PRT-14425)	Digi-Key (1568-1709-ND)	1	1.50	1.50
Flexible Qwiic Cable – 500 mm	SparkFun (PRT-17257)	Digi-Key (1568-PRT-17257-ND)	2	2.38	4.76
Flexible Qwiic Cable – 50 mm	SparkFun (PRT-17260)	Digi-Key (1568-PRT-17260-ND)	1	1.06	1.06
2.5 mm Pitch 2-pin Cable Matching Pair	Adafruit (4872)	Digi-Key (1528-4872-ND)	18	0.95	17.10
Hook-up Wire Spool Set - 22AWG Stranded-Core – 10 × 25ft	Adafruit (3175)	Digi-Key (1528-1746-ND)	1	29.95	29.95
Solid-Core Wire Spool - 25 ft - 22AWG - Red	Adafruit (288)	Digi-Key (1528-1750-ND)	1	2.95	2.95
Kable Kontrol Heat Shrink Tubing - 2:1 Polyolefin – 3/16” - 10’ Various Colors	Cables Ties And More (HS357-S10)	Cables Ties And More (HS357-S10)	6	5.97	35.82
Kable Kontrol Heat Shrink Tubing - 2:1 Polyolefin – 1/8” - 10’ Black	Cables Ties And More (HS355-S10-BLACK)	Cables Ties And More (HS355-S10-BLACK)	1	5.50	5.50
Translucent Clear PRO Series PETG Filament - 2.85 mm (1 kg; or equivalent)	MatterHackers (M-A9A-GJF6)	MatterHackers (M-A9A-GJF6)	1	55.00	55.00
Black PRO Series PETG Filament - 2.85 mm (1 kg; or equivalent)	MatterHackers (M-M82-QGPE)	MatterHackers (M-M82-QGPE)	2	55.00	110
Purple MH Build Series PLA Filament - 2.85 mm (1 kg; or equivalent)	MatterHackers(M-ZR7-0EQU)	MatterHackers(M-ZR7-0EQU)	1	20.87	20.87
Food Safe Clear Epoxy Resin 1 Gallon	ZDSpoxy (B07VZBC6CZ)	Amazon (727040490164)	1	89.98	89.98
One-Hole Rubber Stoppers Size 5.5 – Pack of 28	Grainger (16ZD52)	Grainger (16ZD52)	2	22.71	45.42
1.5” Straight Sipper Tube (custom order request)	Ancare (OT-99)	Ancare (OT-99)	36	1.77	63.72
¼” Conductive Copper Tape	Bertech (CFT-1/4)	Digi-Key (4393-CFT-1/4-ND)	1	11.84	11.84
				Total	596.66
				Average Price per Cage	33.15

Prices are accurate as of November 2022.

## Synthesis

Reviewing Editor: Mark Laubach, American University Decisions are customarily a result of the Reviewing Editor and the peer reviewers coming together and discussing their recommendations until a consensus is reached. When revisions are invited, a fact-based synthesis statement explaining their decision and outlining what is needed to prepare a revision will be listed below. The following reviewer(s) agreed to reveal their identity: Melissa Herman, Alexxai Kravitz.

Two experts reviewed your paper and felt that revisions are needed before it can be considered for publication. Their comments are given below in full. Please revise to address all points that were raised and resubmit your paper. Thank you for sending it to eNeuro!

Reviewer 1

This report by Petersen et al., describes a new model system for tracking voluntary liquid consumption in mice using a capacitance sensor-based open source platform, Lick Instance Quantifier Home cage Device (LIQ HD). The authors validate their LIQ HD system using two bottle choice sucrose, quinine, and ethanol drinking at a range of concentrations and present data demonstrating that LIQ HD produces accurate and consistent lick microstructure behavior that corresponds to total fluid consumption and replicates previous reports on drinking behavior. In addition, the system offers multi-unit control from a single Arduino and the ability to perform more prolonged drinking experiments with minimal disturbance to the animal. This system represents a significant new tool in the study of drinking behavior which has wide relevance to the field of neuroscience in the context of alcohol drinking but also manipulations related to motivated behavior as measured by sucrose and/or quinine consumption. I only have a few comments/questions about the background rationale and some minor issues with the experimental validation.

1. There is a strong emphasis on the importance of drinking microstructure and the need to be able to measure individual ‘licks’ in drinking bouts, however the background provides little rationale for why this behavior is important. The authors may want to consider providing examples of changes in drinking behavior that necessitate the need for the enhanced precision involved in the LIQ HD system

2. The authors report the use of adult female C57BL/6J mice, however no use of male mice is noted. As most studies endeavor to include both males and females ad there are notable sex differences in some drinking behaviors, the authors may want to consider including data form male mice or list this is a limitation in their validation.

3. The authors report that “Bout microstructure did not significantly differ between the light and dark phase; thus, bout analysis is binned in 24-hour bins”. Variability in drinking microstructure by light cycle is important information and the authors may want to consider including examples of the lack of difference between bout microstructure (total lick #, lick duration, etc..) in the text.

Minor

- Figure 1 A and Extended Figure 1-1A might benefit from labeling.

- There is a typo in the first paragraph of the Results section where the word ‘complied’ is used in place of compiled.

Reviewer 2

This paper by Petersen and colleague describes a novel home-cage compatible 2-bottle lick detecting device. The design is brilliant and they show extremely nice validation data of it, especially the benefits of using capacitive sensing vs. beam-breaks for detecting licking behavior in mice. This is a welcome addition to the open-source behavioral world! That said, I noted some minor edits that I believe should be made before publication.

1. I was excited to see the author’s Github and the efforts they made towards dissemination and creating a device that others could use. This was a major strength of the paper.

2. Full disclosure, I am Lex Kravitz, one of the authors of the Godynyuk et al paper that also described a sipper device. I feel like this paper was unfairly critical of our device in a couple places. Examples:

- In two places the authors say that having a single microcontroller for each sipper device limits scalability. I believe the opposite - in my opinion a distributed system with individual microcontrollers for each device is more scalable. In fact, this is the design principle for most modern electronic systems. In a recent paper (Slivicki et al, Addiction Biology 2022, which could be cited here too) we deployed 30 of our sipper devices equipped with wireless radios to transmit data in real time to a cloud dashboard. Overall I don’t think either system design is inherently scalable or not, but don’t think it’s right to say that putting microcontroller on each device is not scalable.

- The authors start their paper with a comparison to the Godynyuk/Frie designs and note issues they encountered such as mice destroying the sensors and the devices needing many repairs. I think this section gives the impression that the Godynyuk/Frie designs are the cause of these issues. However, the authors completely re-designed our device for this experiment, changing the 3D design, the electronics, and even the bottles used in it. I therefore didn’t think this was a fair comparison to our device and thought that the extensive modifications they made to our design likely induced the issues they experienced. However the section read like these were issues inherent to our design. I would like them to reconsider how this section is written, and distance it from our devices, as that was not what was tested here. I am not totally sure this comparison is useful, as it seems like they had many issues with their modified beam-break device.

- I do think they show the value of touch sensing beautifully, and their paper should focus on that advancement vs. the comparison with existing solutions.

3. I was concerned about the 3D printed bottles, and in particular 1) whether the epoxy is food-safe, and 2) how the authors clean them out and sterilize them - in my experience bottles with sucrose solutions can grow bacteria and/or fungi that is hard to clean. Can the authors discuss this? Is there a reason a commercial plastic or glass bottle could not have been used? I found this to be the most problematic part of the build, and I think a suitable commercial bottle would make this build much nicer by addressing potential issues of cleaning and sterilizing 3D printed bottles.

4. I was curious whether the device can detect licks that occur simultaneously within different cages? Or if one sipper touch sensor is triggered does it prohibit other sippers from registering?

5. I would have liked to see an example of the data output file, as I was confused about this in my reading of the paper and Github site. On one hand the authors were recording every lick, and they reported things like inter-lick interval in their paper, but from the Github I got the sense that the data was saved in 1-minute bins and measures like inter-lick interval were not available to users. Including a sample data file on the Github site (or with the extended data) would be really helpful for a user to get a sense of what the device produces.
