# Supplementary figures and images for: LIQ HD (Lick Instance Quantifier Home Cage Device): An Open-Source Tool for Recording Undisturbed Two-Bottle Drinking Behavior in a Home Cage Environment
Source: eNeuro. 2023 Apr 12;10(4):ENEURO.0506-22.2023. doi: 10.1523/ENEURO.0506-22.2023 (PMC10112549; doi:10.1523/ENEURO.0506-22.2023)

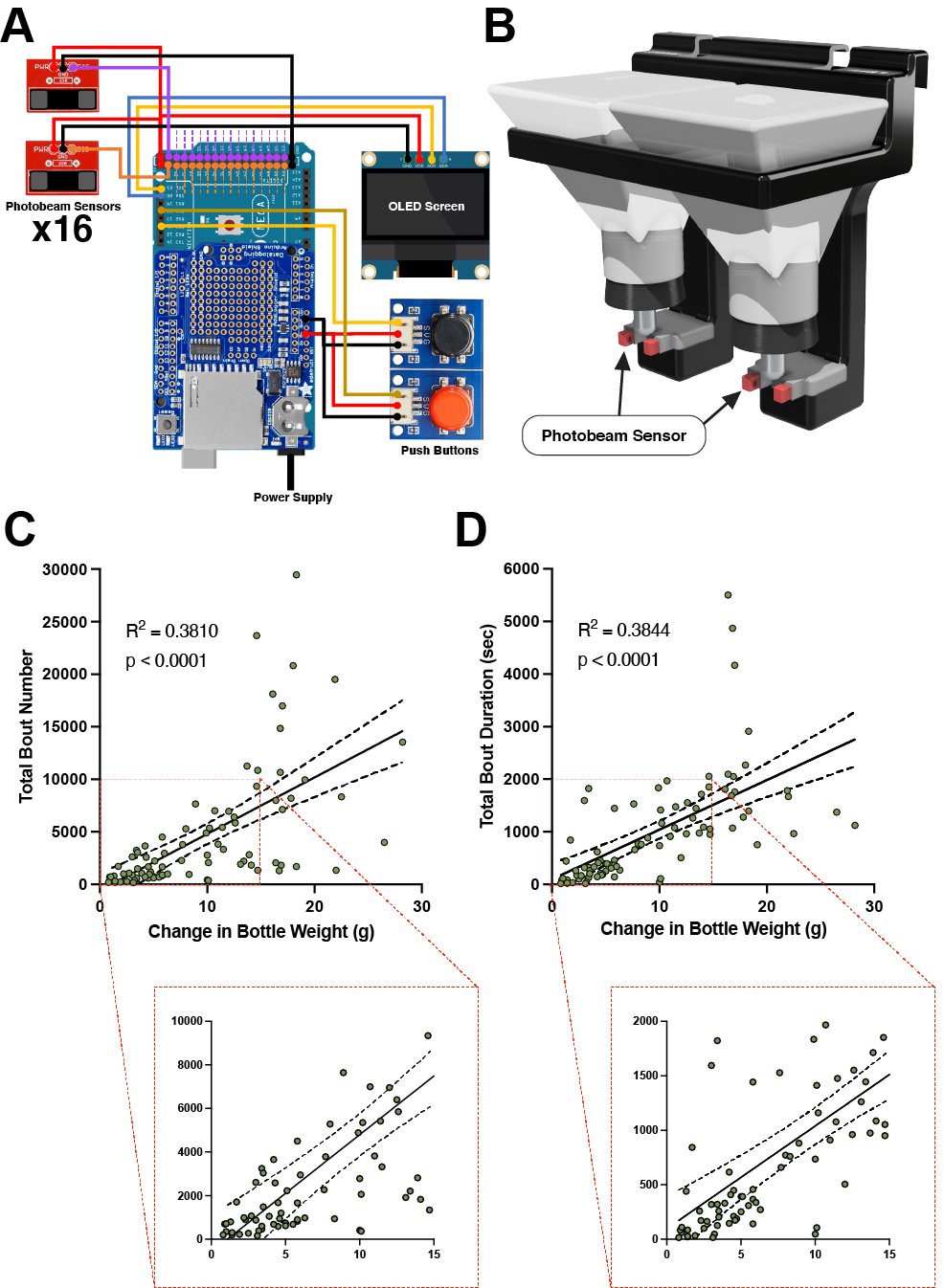

Supplement: Extended Data Figure 1-1 — Infrared photobeam-based two-bottle choice device design and validation. A, Electronic parts and wiring diagram for the beam-break system. B, 3D rendering of beam-break device, including 3D-printed components, rubber stoppers and sippers, and photobeam sensors (red). C, Correlation between total bout number and change in bottle weight for each recording period. D, Correlation between total bout duration and change in bottle weight for each recording period. Solid lines represent fitted simple linear regression models, and dashed lines denote 95% confidence intervals. Download Figure 1-1, TIF file. [file enu-eN-NWR-0506-22-s01.tif]

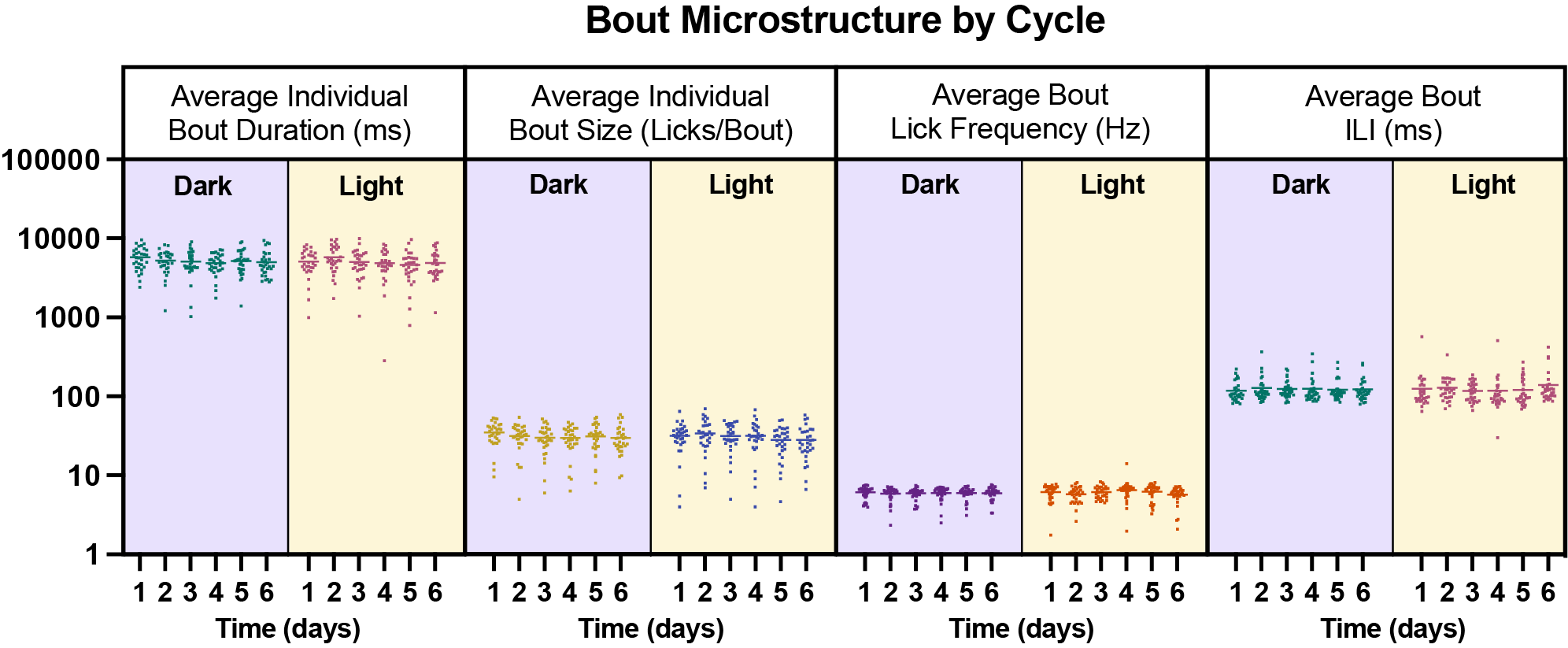

Supplement: Extended Data Figure 4-1 — Bout microstructure does not significantly differ between the light and dark cycle. A total of 16 mice were given access to two water bottles for a one-week recording period. The data for the 32 bottles were pooled and binned into 12-h bins to determine differences in bout microstructure between the light and dark cycle. Mixed-effects models with the Geisser–Greenhouse correction using a compound symmetry covariance matrix and fit using restricted maximum likelihood (REML) revealed no significant main effect of light cycle for the average individual bout duration (F(1,31) = 0.8731, p = 0.3573), average individual bout size (F(1,31) = 0.2621, p = 0.6123), average bout lick frequency (F(1,31) = 1.019, p = 0.3206), or average ILI (F(1,31) = 0.1218, p = 0.7294; N = 16 mice, n = 32 bottles). Download Figure 4-1, TIF file. [file enu-eN-NWR-0506-22-s03.tif]
